# Supplementary material for: Anodal transcranial direct current stimulation reduces collinear lateral inhibition in normal peripheral vision
Source: PLoS One. 2020 May 6;15(5):e0232276. doi: 10.1371/journal.pone.0232276 (PMC7202594; doi:10.1371/journal.pone.0232276)
Supplement: S1 Table — (DOCX) [file pone.0232276.s001.docx]

Supplementary Table 1: Mean contrast threshold values (db) and SD of reversals for each participant in 2λ condition

|  |  | 2λ | | | | | | | |
| --- | --- | --- | --- | --- | --- | --- | --- | --- | --- |
|  |  | *BL_A* | *DS_A* | *PS5_A* | *PS30_A* | *BL_S* | *DS_S* | *PS5_S* | *PS30_S* |
| S1 | Avg. threshold | 0.100 | 0.088 | 0.144 | 0.100 | 0.132 | 0.108 | 0.092 | 0.084 |
|  | SD of reversals | 0.047 | 0.041 | 0.089 | 0.032 | 0.054 | 0.048 | 0.018 | 0.043 |
| S2 | Avg. threshold | 0.249 | 0.177 | 0.229 | 0.223 | 0.235 | 0.208 | 0.213 | 0.213 |
|  | SD of reversals | 0.032 | 0.034 | 0.028 | 0.056 | 0.045 | 0.064 | 0.051 | 0.047 |
| S3 | Avg. threshold | 0.765 | 0.400 | 0.453 | 0.553 | 0.343 | 0.443 | 0.447 | 0.500 |
|  | SD of reversals | 0.084 | 0.089 | 0.024 | 0.043 | 0.053 | 0.088 | 0.059 | 0.042 |
| S4* | Avg. threshold | 0.535 | 0.210 | 0.360 | 0.280 | 0.280 | 0.377 | 0.277 | 0.227 |
|  | SD of reversals | 0.019 | 0.012 | 0.016 | 0.016 | 0.018 | 0.046 | 0.056 | 0.024 |
| S5 | Avg. threshold | 0.435 | 0.095 | 0.220 | 0.165 | 0.270 | 0.305 | 0.185 | 0.120 |
|  | SD of reversals | 0.025 | 0.019 | 0.028 | 0.034 | 0.026 | 0.030 | 0.100 | 0.037 |
| S6 | Avg. threshold | 0.385 | 0.265 | 0.290 | 0.225 | 0.393 | 0.267 | 0.187 | 0.260 |
|  | SD of reversals | 0.025 | 0.044 | 0.012 | 0.019 | 0.031 | 0.042 | 0.031 | 0.020 |
| S7 | Avg. threshold | 0.277 | 0.223 | 0.169 | 0.214 | 0.385 | 0.375 | 0.355 | 0.450 |
|  | SD of reversals | 0.069 | 0.063 | 0.063 | 0.043 | 0.066 | 0.038 | 0.019 | 0.012 |
| S8 | Avg. threshold | 0.420 | 0.389 | 0.349 | 0.349 | 0.240 | 0.205 | 0.235 | 0.345 |
|  | SD of reversals | 0.026 | 0.020 | 0.061 | 0.047 | 0.016 | 0.019 | 0.050 | 0.038 |
| S9 | Avg. threshold | 0.525 | 0.415 | 0.490 | 0.410 | 0.405 | 0.443 | 0.513 | 0.408 |
|  | SD of reversals | 0.034 | 0.034 | 0.026 | 0.012 | 0.062 | 0.038 | 0.123 | 0.030 |
| S10 | Avg. threshold | 0.212 | 0.240 | 0.200 | 0.168 | 0.343 | 0.275 | 0.295 | 0.350 |
|  | SD of reversals | 0.018 | 0.024 | 0.079 | 0.041 | 0.025 | 0.064 | 0.082 | 0.105 |
| S11 | Avg. threshold | 0.285 | 0.200 | 0.285 | 0.205 | 0.385 | 0.310 | 0.305 | 0.300 |
|  | SD of reversals | 0.038 | 0.037 | 0.019 | 0.034 | 0.082 | 0.062 | 0.019 | 0.059 |
| S12 | Avg. threshold | 0.344 | 0.248 | 0.336 | 0.328 | 0.550 | 0.375 | 0.405 | 0.420 |
|  | SD of reversals | 0.059 | 0.059 | 0.055 | 0.050 | 0.012 | 0.019 | 0.053 | 0.028 |
| S13 | Avg. threshold | 0.504 | 0.420 | 0.344 | 0.388 | 0.437 | 0.417 | 0.277 | 0.477 |
|  | SD of reversals | 0.074 | 0.077 | 0.022 | 0.052 | 0.070 | 0.052 | 0.021 | 0.031 |
| **Overall Mean ± SD** | | **0.387±0.173** | **0.259±0.115** | **0.297±0.105** | **0.277±0.124** | **0.338±0.107** | **0.316±0.102** | **0.291±0.116** | **0.319±0.133** |

Abbreviations: BL – baseline; DS – during stimulation; PS5 and PS30 – post-stimulation after 5min and 30min respectively. A – active; S – Sham. *denotes the participant with a staircase removed due to unreliability.
