# Supplementary material for: Anodal transcranial direct current stimulation reduces collinear lateral inhibition in normal peripheral vision
Source: PLoS One. 2020 May 6;15(5):e0232276. doi: 10.1371/journal.pone.0232276 (PMC7202594; doi:10.1371/journal.pone.0232276)
Supplement: S2 Table — (DOCX) [file pone.0232276.s002.docx]

Supplementary Table 2: Mean contrast threshold values (db) and SD of reversals for each participant in 6λ condition

|  |  | 6λ | | | | | | | |
| --- | --- | --- | --- | --- | --- | --- | --- | --- | --- |
|  |  | *BL_A* | *DS_A* | *PS5_A* | *PS30_A* | *BL_S* | *DS_S* | *PS5_S* | *PS30_S* |
| S1 | Avg. threshold | 0.028 | 0.024 | 0.028 | 0.064 | 0.018 | 0.028 | 0.033 | 0.020 |
|  | SD of reversals | 0.011 | 0.017 | 0.018 | 0.062 | 0.023 | 0.021 | 0.028 | 0.019 |
| S2 | Avg. threshold | 0.037 | 0.080 | 0.075 | 0.069 | 0.033 | 0.043 | 0.040 | 0.043 |
|  | SD of reversals | 0.035 | 0.060 | 0.063 | 0.034 | 0.032 | 0.033 | 0.067 | 0.029 |
| S3 | Avg. threshold | 0.409 | 0.423 | 0.378 | 0.383 | 0.140 | 0.368 | 0.355 | 0.278 |
|  | SD of reversals | 0.053 | 0.029 | 0.033 | 0.117 | 0.075 | 0.021 | 0.056 | 0.039 |
| S4 | Avg. threshold | 0.289 | 0.311 | 0.186 | 0.274 | 0.150 | 0.310 | 0.190 | 0.048 |
|  | SD of reversals | 0.066 | 0.045 | 0.085 | 0.025 | 0.035 | 0.082 | 0.078 | 0.028 |
| S5 | Avg. threshold | 0.058 | 0.086 | 0.093 | 0.063 | 0.370 | 0.128 | 0.228 | 0.118 |
|  | SD of reversals | 0.036 | 0.082 | 0.117 | 0.069 | 0.060 | 0.059 | 0.063 | 0.132 |
| S6 | Avg. threshold | 0.145 | 0.230 | 0.355 | 0.145 | 0.186 | 0.155 | 0.260 | 0.328 |
|  | SD of reversals | 0.019 | 0.012 | 0.066 | 0.019 | 0.063 | 0.093 | 0.034 | 0.043 |
| S7 | Avg. threshold | 0.026 | 0.131 | 0.069 | 0.033 | 0.045 | 0.063 | 0.050 | 0.086 |
|  | SD of reversals | 0.015 | 0.169 | 0.047 | 0.024 | 0.066 | 0.071 | 0.051 | 0.061 |
| S8 | Avg. threshold | 0.188 | 0.055 | 0.253 | 0.163 | 0.070 | 0.077 | 0.085 | 0.097 |
|  | SD of reversals | 0.164 | 0.033 | 0.038 | 0.105 | 0.039 | 0.098 | 0.050 | 0.091 |
| S9 | Avg. threshold | 0.125 | 0.130 | 0.115 | 0.100 | 0.280 | 0.405 | 0.178 | 0.185 |
|  | SD of reversals | 0.026 | 0.024 | 0.030 | 0.024 | 0.057 | 0.100 | 0.048 | 0.058 |
| S10 | Avg. threshold | 0.116 | 0.172 | 0.084 | 0.108 | 0.140 | 0.278 | 0.255 | 0.283 |
|  | SD of reversals | 0.038 | 0.127 | 0.043 | 0.175 | 0.034 | 0.140 | 0.023 | 0.063 |
| S11 | Avg. threshold | 0.015 | 0.187 | 0.170 | 0.265 | 0.265 | 0.070 | 0.370 | 0.318 |
|  | SD of reversals | 0.019 | 0.031 | 0.012 | 0.034 | 0.085 | 0.100 | 0.176 | 0.063 |
| S12 | Avg. threshold | 0.093 | 0.098 | 0.108 | 0.073 | 0.120 | 0.048 | 0.115 | 0.053 |
|  | SD of reversals | 0.054 | 0.055 | 0.111 | 0.084 | 0.030 | 0.026 | 0.195 | 0.032 |
| S13 | Avg. threshold | 0.180 | 0.090 | 0.127 | 0.117 | 0.158 | 0.148 | 0.228 | 0.195 |
|  | SD of reversals | 0.075 | 0.049 | 0.037 | 0.043 | 0.125 | 0.076 | 0.122 | 0.048 |
| **Overall Mean ± SD** | | **0.131±0.115** | **0.155±0.112** | **0.157±0.109** | **0.152±0.104** | **0.143±0.104** | **0.163±0.132** | **0.183±0.114** | **0.158±0.113** |

Abbreviations: BL – baseline; DS – during stimulation; PS5 and PS30 – post-stimulation after 5min and 30min respectively. A – active; S – Sham.
